# Dual roles of jasmonate in adventitious rooting

**DOI:** 10.1093/jxb/erab378

**Published:** 2021-10-26

**Authors:** Xuan Pan, Zhengfei Yang, Lin Xu

**Affiliations:** 1 National Key Laboratory of Plant Molecular Genetics, CAS Center for Excellence in Molecular Plant Sciences, Institute of Plant Physiology and Ecology, Chinese Academy of Sciences, 300 Fenglin Road, Shanghai 200032, China; 2 University of Chinese Academy of Sciences, 19A Yuquan Road, Beijing, 100049, China; 3 College of Life Sciences, Shanghai Normal University, Shanghai 200234, China

**Keywords:** Adventitious root, Arabidopsis, cytokinin, jasmonate

## Abstract

This article comments on:

**Dob A, Lakehal A, Novak O, Bellini C.** 2021. Jasmonate inhibits adventitious root initiation through repression of *CKX1* and activation of RAP2.6L transcription factor in Arabidopsis. Journal of Experimental Botany **72**, 7107–7118.

**Plants may initiate adventitious root growth from non-root organs (Bellini *et al.*, 2014; Druege *et al.*, 2019). In *Arabidopsis thaliana*, adventitious roots form either from hypocotyls, contributing to the root system architecture, or from wounded organs during regeneration (Bellini *et al.*, 2014; Xu, 2018). Jasmonate (JA), a wound-induced hormone, regulates adventitious rooting from hypocotyls and wounded organs. Interestingly, JA plays opposite adventitious rooting-associated regulatory roles at different stages and from different sources through crosstalk with multiple hormones. In this issue of the *Journal of Experimental Botany*, Dob *et al.* (2021) show that the crosstalk between JA and cytokinin regulates adventitious rooting from hypocotyls.**

Adventitious roots are initiated from non-root organs. In Arabidopsis, hypocotyls can form adventitious roots to replenish the root system architecture, and wounded regions of detached leaves can also form adventitious roots, termed *de novo* root regeneration. Auxin is the central hormone in rooting (Thimann *et al.*, 1934). In each of these Arabidopsis adventitious rooting processes, auxin is the key hormone that controls the initiation of the adventitious root primordium. The auxin signalling pathway can directly target root cell fate-controlling genes, such as *WUSCHEL RELATED HOMEOBOX 11* (*WOX11*) and *LATERAL ORGAN BOUNDARIES DOMAIN 16* (*LBD16*) (Liu *et al.*, 2014; Sheng *et al.*, 2017), as well as targeting the *Gretchen Hagen3 (GH3.3*, *GH3.5*, and *GH3.6*) genes involved in JA homeostasis (Gutierrez *et al.*, 2012; Lakehal et al., 2019a). Other hormones, such as JA, cytokinin, ethylene, gibberellin, and abscisic acid, also play roles in regulating the efficiency of adventitious rooting (reviewed in Druege *et al.*, 2019). In addition, many hormones are sensitive to, and regulated by, environmental signals, thereby linking environmental conditions to root organogenesis.

## JA serves as a negative regulator of adventitious rooting from hypocotyls

In this issue, Dob *et al*. analysed the crosstalk between JA and cytokinin during the inhibition of adventitious rooting from Arabidopsis hypocotyls. Cytokinin has inhibitory roles in adventitious rooting from hypocotyls. The authors found that the cytokinin free base content decreases during adventitious rooting from hypocotyls, which might be caused by the down-regulation of cytokinin biosynthesis and the up-regulation of cytokinin inactivation pathways. They further show that the JA–*MYC2* signalling pathway represses the expression of *CYTOKININ OXIDASE/DEHYDROGENASE1* (*CKX1*), which is responsible for cytokinin degradation, thereby inhibiting adventitious root formation. Additionally, JA and cytokinin may synergistically activate the *RELATED to APETALA2.6 LIKE (RAP2.6L*) [also known as *ETHYLENE RESPONSE FACTOR* (*ERF*) *113*] transcription factor gene, a negative regulator of adventitious rooting. Overall, the crosstalk between JA and cytokinin is essential for the negative regulation of root organogenesis from hypocotyls.

Previous studies from this group also suggest that JA inhibits adventitious rooting from hypocotyls through multiple molecular pathways. The expression of *ERF115* is activated by JA, which promotes cytokinin biosynthesis to inhibit adventitious rooting (Lakehal *et al.*, 2020). In addition, JA promotes the expression of the *DIOXYGENASE FOR AUXIN OXIDATION 1* (*DAO1*) gene, which regulates the feedback of the auxin–JA crosstalk (Lakehal *et al.*, 2019b). Therefore, JA, auxin, and cytokinin are involved in the complex regulatory network controlling adventitious rooting from hypocotyls.

## JA serves as a positive regulator of adventitious rooting from detached leaves

In addition to its inhibitory role in adventitious rooting from hypocotyls, JA functions as a positive regulator promoting adventitious root organogenesis. Detached leaves can regenerate adventitious roots upon wounding, and JA has an important promotive role in this regenerative process (Zhang *et al.*, 2019). The JA level is highly induced in detached leaves within 10 min of wounding. The JA signalling pathway then directly activates *ERF109*, which in turn promotes the expression of *ANTHRANILATE SYNTHASE α1* (*ASA1*), a tryptophan biosynthetic gene. Tryptophan is the amino acid precursor that can be converted to form auxin. The quick activation of *ASA1* by ERF109 is dependent on the deposition of H3 lysine 36 trimethylation (H3K36me3) on the *ASA1* locus by SET DOMAIN GROUP8 (SDG8) histone methyltransferase. Thus, JA may act as a wound hormone to promote adventitious rooting by up-regulating the auxin level. However, constant long-term JA signalling is harmful to root organogenesis. The leaf explant turns off the JA signal partly through the interaction of ERF109 with JASMONATE-ZIM-DOMAIN (JAZ) proteins to block the ERF109 activity.

The JA–ERF module is also regulated by the age developmental pathway in Arabidopsis (Ye *et al.*, 2020). As plants age, the adventitious root formation capacity from detached organs gradually decreases. In Arabidopsis, the miRNA156 (miR156)–*SQUAMOSA PROMOTER BINDING PROTEIN-LIKE* (*SPL*) pathway is the key regulator of ageing, in which miR156 directly targets the mRNAs of the *SPL* transcription factor genes for mRNA degradation. With ageing, the miR156 level decreases, while the *SPL* mRNA level increases. In adventitious rooting from leaf explants, *SPL2*, *10*, and *11* are the key negative regulatory genes. They negatively regulate *ERF109* and another *ERF* gene, *ABSCISIC ACID REPRESSOR1* (*ABR1*), resulting in the restriction of auxin production for rooting. Therefore, the JA–ERF pathway is highly sensitive to the leaf explant age.

## Perspectives

The roles of JA, both positive and negative, in adventitious rooting have been observed in Arabidopsis and many other plant species (Ahkami *et al.*, 2009; Fattorini *et al*., 2009, 2018; Gutierrez *et al.*, 2012; Agulló-Antón *et al.*, 2014; Lischweski *et al.*, 2015; Park *et al.*, 2019; Lakehal *et al.*, 2019b; Zhang *et al.*, 2019; Alallaq *et al.*, 2020). Presently, it is hypothesized that JA serves a dual role in adventitious rooting (summarized in Box 1). On the one hand, JA inhibits root organogenesis through crosstalk with cytokinin and auxin. On the other hand, JA serves as a wound signal that promotes auxin production to activate rooting. Therefore, the rigorously controlled JA level and its spatial pattern may be important for adventitious root formation.

**Box 1. The molecular network of jasmonate (JA) and other hormones in adventitious rooting.**

JA plays dual roles in the regulation of adventitious rooting. During adventitious rooting from Arabidopsis hypocotyls, there is crosstalk among JA, auxin, and cytokinin, thereby forming a molecular network that inhibits root organogenesis. During adventitious rooting from detached leaf explants, JA acts as a wound signal that up-regulates auxin biosynthesis to promote root organogenesis and is regulated by the ageing pathway. The opposite roles of JA in the two adventitious rooting processes may indicate that JA acts in a strict spatial and temporal manner to regulate adventitious root formation.

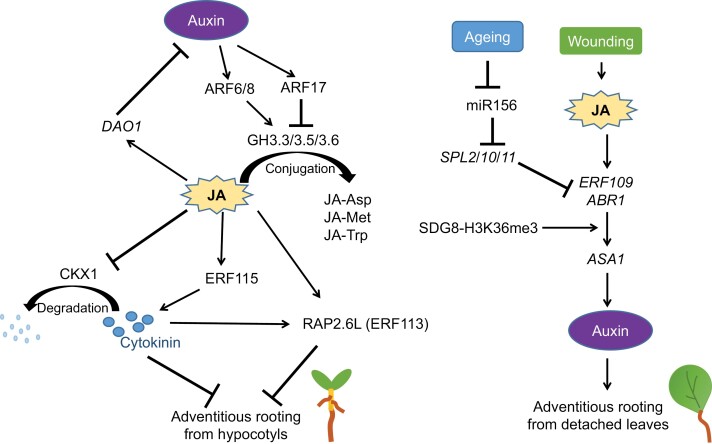

Many important questions regarding the role of JA in adventitious rooting remain unanswered. For example, in adventitious rooting from detached leaf explants, a constant JA treatment blocks the rooting process (Zhang *et al.*, 2019). Whether the inhibitory role of JA in rooting from detached leaves and its role in rooting from hypocotyls share the same mechanisms is not clear. Additionally, the endogenous JA levels must be rigorously controlled in different stages of adventitious rooting and in different organs that produce adventitious roots. What is the mechanism that spatially and temporally regulates JA levels? Further studies on JA in the adventitious rooting of Arabidopsis and other species will shed light on the complex regulatory network of root organogenesis.

## References

[CIT0001] Agulló-Antón MÁ , Ferrández-Ayela A, Fernández-García N, Nicolás C, Albacete A, Pérez-Alfocea F, Sánchez-Bravo J, Pérez-Pérez JM, Acosta M. 2014. Early steps of adventitious rooting: morphology, hormonal profiling and carbohydrate turnover in carnation stem cuttings. Physiologia Plantarum 150, 446–462. 24117983 10.1111/ppl.12114

[CIT0002] Ahkami AH , Lischewski S, Haensch KT, et al. 2009. Molecular physiology of adventitious root formation in Petunia hybrida cuttings: involvement of wound response and primary metabolism. The New phytologist 181, 613–625. 19076299 10.1111/j.1469-8137.2008.02704.x

[CIT0003] Alallaq S , Ranjan A, Brunoni F, Novák O, Lakehal A, Bellini C. 2020. Red Light Controls Adventitious Root Regeneration by Modulating Hormone Homeostasis in Picea abies Seedlings. Frontiers in Plant Science 11, 586140. 33014006 10.3389/fpls.2020.586140 PMC7509059

[CIT0004] Bellini C , Pacurar DI, Perrone I. 2014. Adventitious roots and lateral roots: similarities and differences. Annual Review of Plant Biology 65, 639–666. 10.1146/annurev-arplant-050213-035645 24555710

[CIT0005] Dob A , Lakehal A, Novak O, Bellini C. 2021. Jasmonate inhibits adventitious root initiation through repression of CKX1 and activation of RAP2.6L transcription factor in Arabidopsis. Journal of Experimental Botany 72, 7107–7118 10.1093/jxb/erab358 PMC8547155 34329421

[CIT0006] Druege U , Hilo A, Pérez-Pérez JM, Klopotek Y, Acosta M, Shahinnia F, Zerche S, Franken P, Hajirezaei MR. 2019. Molecular and physiological control of adventitious rooting in cuttings: phytohormone action meets resource allocation. Annals of Botany 123, 929–949. 30759178 10.1093/aob/mcy234 PMC6589513

[CIT0007] Fattorini L , Falasca G, Kevers C, Rocca LM, Zadra C, Altamura MM. 2009. Adventitious rooting is enhanced by methyl jasmonate in tobacco thin cell layers. Planta 231, 155–168. 19885676 10.1007/s00425-009-1035-y

[CIT0008] Fattorini L , Hause B, Gutierrez L, Veloccia A, Della Rovere F, Piacentini D, Falasca G, Altamura MM. 2018. Jasmonate promotes auxin-induced adventitious rooting in dark-grown Arabidopsis thaliana seedlings and stem thin cell layers by a cross-talk with ethylene signalling and a modulation of xylogenesis. BMC Plant Biology 18, 182. 30189848 10.1186/s12870-018-1392-4 PMC6127917

[CIT0009] Gutierrez L , Mongelard G, Floková K, et al. 2012. Auxin controls Arabidopsis adventitious root initiation by regulating jasmonic acid homeostasis. The Plant Cell 24, 2515–2527. 22730403 10.1105/tpc.112.099119 PMC3406919

[CIT0010] Lakehal A , Chaabouni S, Cavel E, et al. 2019a. A Molecular Framework for the Control of Adventitious Rooting by TIR1/AFB2-Aux/IAA-Dependent Auxin Signaling in Arabidopsis. Molecular Plant 12, 1499–1514. 31520787 10.1016/j.molp.2019.09.001

[CIT0011] Lakehal A , Dob A, Novák O, Bellini C. 2019 *b*. A DAO1-mediated circuit controls auxin and jasmonate crosstalk robustness during adventitious root initiation in Arabidopsis. International Journal of Molecular Sciences 20, 4428. 10.3390/ijms20184428 PMC6769753 31505771

[CIT0012] Lakehal A , Dob A, Rahneshan Z, Novák O, Escamez S, Alallaq S, Strnad M, Tuominen H, Bellini C. 2020. ETHYLENE RESPONSE FACTOR 115 integrates jasmonate and cytokinin signaling machineries to repress adventitious rooting in Arabidopsis. The New phytologist 228, 1611–1626. 32634250 10.1111/nph.16794

[CIT0013] Lischweski S , Muchow A, Guthörl D, Hause B. 2015. Jasmonates act positively in adventitious root formation in petunia cuttings. BMC Plant Biology 15, 229. 26394764 10.1186/s12870-015-0615-1 PMC4579608

[CIT0014] Liu J , Sheng L, Xu Y, Li J, Yang Z, Huang H, Xu L. 2014. WOX11 and 12 are involved in the first-step cell fate transition during de novo root organogenesis in Arabidopsis. The Plant Cell 26, 1081–1093. 24642937 10.1105/tpc.114.122887 PMC4001370

[CIT0015] Park OS , Bae SH, Kim SG, Seo PJ. 2019. JA-pretreated hypocotyl explants potentiate de novo shoot regeneration in Arabidopsis. Plant Signaling & Behavior 14, 1618180. 31094274 10.1080/15592324.2019.1618180 PMC6619942

[CIT0016] Sheng L , Hu X, Du Y, Zhang G, Huang H, Scheres B, Xu L. 2017. Non-canonical WOX11-mediated root branching contributes to plasticity in Arabidopsis root system architecture. Development 144, 3126–3133. 28743799 10.1242/dev.152132 PMC5611959

[CIT0017] Thimann KV , Went FW. 1934. On the chemical nature of the rootforming hormone. Proceedings of the Koninklinjke Nederlandse Akademie van Wetensschappen Series C 37, 456–459.

[CIT0018] Xu L . 2018. De novo root regeneration from leaf explants: wounding, auxin, and cell fate transition. Current Opinion in Plant Biology 41, 39–45. 28865805 10.1016/j.pbi.2017.08.004

[CIT0019] Ye BB , Shang GD, Pan Y, Xu ZG, Zhou CM, Mao YB, Bao N, Sun L, Xu T, Wang JW. 2020. AP2/ERF Transcription Factors Integrate Age and Wound Signals for Root Regeneration. The Plant Cell 32, 226–241. 31649122 10.1105/tpc.19.00378 PMC6961627

[CIT0020] Zhang G , Zhao F, Chen L, et al. 2019. Jasmonate-mediated wound signalling promotes plant regeneration. Nature Plants 5, 491–497. 31011153 10.1038/s41477-019-0408-x

